# The spread of chemical biology into chromatin

**DOI:** 10.1016/j.jbc.2024.107776

**Published:** 2024-09-12

**Authors:** Esmat Hegazi, Tom W. Muir

**Affiliations:** Department of Chemistry, Princeton University, Princeton, New Jersey, USA

**Keywords:** epigenetics, chromatin regulation, protein chemistry, chemical biology, histone post-translational modifications

## Abstract

Understanding the molecular mechanisms underlying chromatin regulation, the complexity of which seems to deepen with each passing year, requires a multidisciplinary approach. While many different tools have been brought to bear in this area, here we focus on those that have emerged from the field of chemical biology. We discuss methods that allow the generation of what is now commonly referred to as “designer chromatin,” a term that was coined by the late C. David (Dave) Allis. Among Dave’s many talents was a remarkable ability to “brand” a nascent area (or concept) such that it was immediately relatable to the broader field. This also had the entirely intentional effect of drawing more people into the area, something that as this brief review attempts to convey has certainly happened when it comes to getting chemists involved in chromatin research.

Contemporary chromatin research continues to exert a considerable gravitational pull, drawing in scientists from fields as diverse as genetics, bioinformatics, biophysics, and chemical biology. This broad attraction stems from the underlying complexity of chromatin biology, the study of which we now realize benefits from a “circle the wagons”-type approach, wherein tools and ideas from disparate areas are productively integrated to reveal fundamental mechanisms. The late Dave Allis, to whom this issue is dedicated, understood the importance of this multidisciplinary approach as much as anyone in the chromatin field. His scientific journey and explorations had revealed just how intricate the fundamental molecular mechanisms attendant to chromatin regulation were likely to be. Consequently, he came to understand that approaching these problems from a single direction, say genetics, would be insufficient to reveal all aspects of the mechanism. Ultimately, Allis was driven by a deep desire to get to the bottom of the various phenomena he had uncovered, something that simply required expertise beyond his formidable skills as a biochemist. Dave was unusually prescient when it came to identifying the types of expertise that were needed to advance the field. As importantly, he could also draw scientists into the area through his ability to distill and synthesize emerging findings in the chromatin biology area into easy-to-grasp concepts that one could rally around and ultimately test. It would be hard to argue that this multidisciplinary approach, of which Allis was a great champion, has not accelerated discoveries in the chromatin field (extensively reviewed in (1, 2, 3, 4)).

In 2011, one of us (T. W. M.) co-authored a perspective article with Dave entitled “*Spreading Chromatin into Chemical Biology*” (5). This piece (hereafter referred to as the ‘2011 perspective’) was written as a “call to arms” for chemical biologists. The goal was to highlight contemporary problems in chromatin biology where we thought the tools of chemical biology, and in particular protein chemistry, could be usefully applied. Our basic thesis was that insights into these various questions would be forthcoming by deploying chemically defined chromatin substrates in which the patterns of histone post-translational modifications (PTMs) are known—in typically lucent fashion Allis referred to such substrates as “designer chromatin.” Now, well over a decade later, we can assess the response to this solicitation. In other words, just how much has chromatin research spread into the field of chemical biology (or *vice versa* depending on one’s perspective)? We will attempt to answer this question by revisiting some of the same problems highlighted in the “2011 perspective” and asking whether the availability of designer chromatin has contributed to our understanding. We stress that this article is not meant to be a comprehensive review of this field, which (spoiler alert) is now extremely large. For instance, we will not discuss how these modified proteins and chromatin substrates are manufactured, noting that this area has been extensively reviewed elsewhere (1, 4, 6, 7). Even within the topics we do cover, our focus is on specific studies that collectively capture the breadth of strategies that have been developed. Again, many excellent reviews cover the specific applications of designer chromatin in great depth (1, 2, 3, 4, 8, 9). Rather, our goal is to illustrate the remarkable progress that has been made and to hopefully pay homage to the pervasive influence (the hidden hand) of our late friend and colleague Dave Allis in the development of this field.

## Designer chromatin

As noted earlier, designer chromatin refers to an *in vitro* preparation of chromatin in which the chemical composition is defined. This should be contrasted with chromatin that is isolated from cells (typically after a sheering and digestion step) which will inevitably be a very complex mixture containing different histone PTM patterns, histone variants, DNA sequences, and DNA methylation levels. While such preparations do have their uses, they do not allow causal relationships to be firmly established between a particular chromatin input state and a given biochemical output. Obtaining such cause-and-effect information is the core justification for generating designer chromatin.

Access to designer chromatin results from the confluence of decades of advances in two areas, namely, (i) *in vitro* reconstitution of mononucleosomes (MNs) and nucleosome arrays (arrays) from constituent histones and DNA templates and, (ii) generation of proteins containing non-coded elements such as PTMs. Since the technical aspects of both these areas have been extensively reviewed elsewhere (7, 9), in this section we focus exclusively on the classes of designer chromatin that can now be made. It is worth stressing that prior to the widespread adoption of designer chromatin most chromatin biochemistry employed modified histone-derived peptides containing PTMs of interest—a practice that goes back several decades to the pioneering studies of Allfrey and Merrifield who like Allis worked at the Rockefeller University (10, 11). Modified peptides are straightforward to synthesize and can provide information on the role of local amino acid sequence on the recognition and activity of the readers, writers and erasers of histone PTMs (4). However, many of these factors engage chromatin through multivalent DNA and histone interactions which cannot be recapitulated with short peptides. Thus, designer chromatin provides a more physiological substrate with which to carry out *in vitro* biochemical and structural investigations.

**Figure 1:**
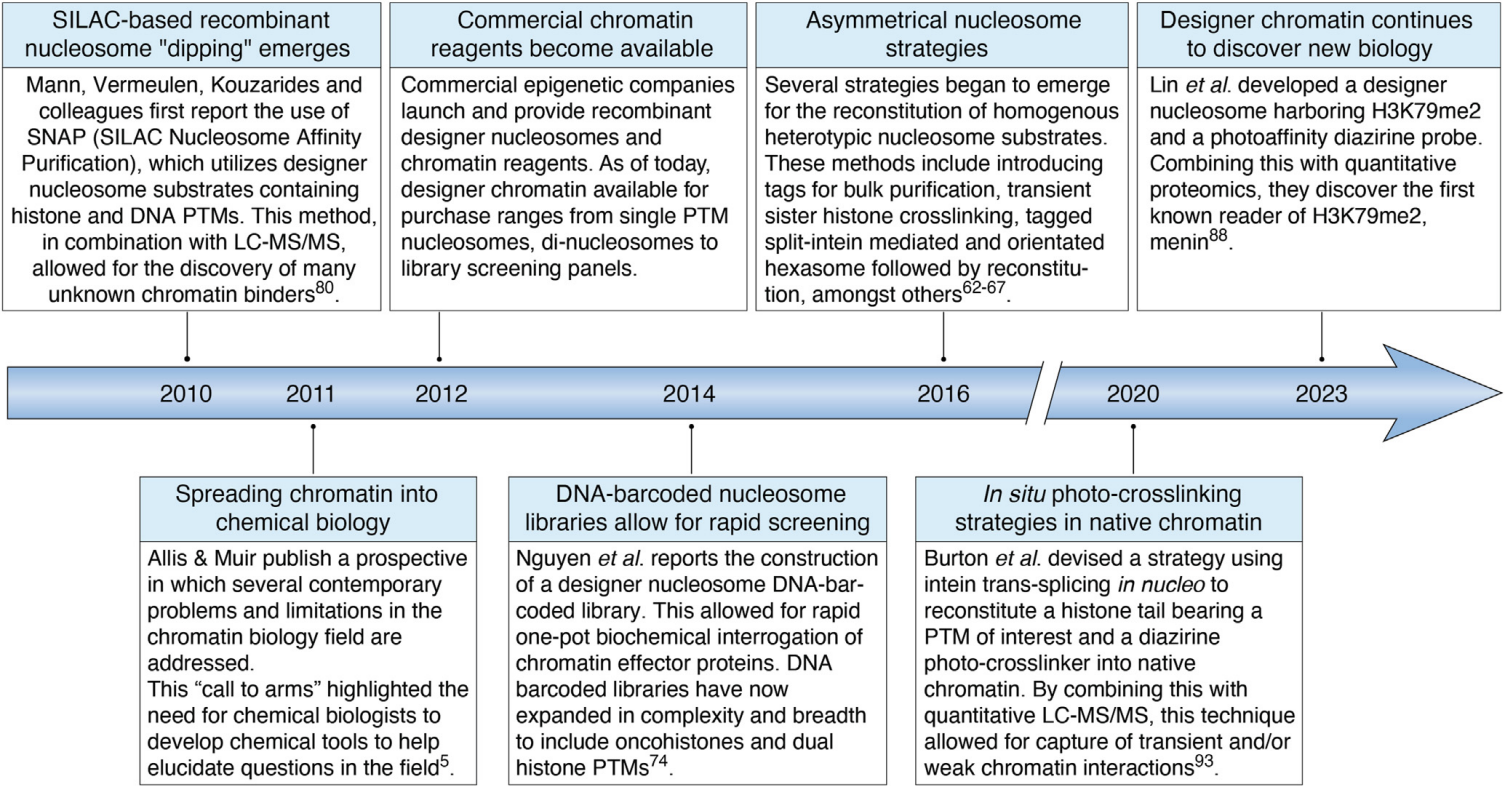
**Timeline highlighting advancements in chemical biology tool development in the study of chromatin since 2010.** Particularly: reagents becoming commercially available, the use of designer chromatin substrates coupled with proteomics for identifying chromatin readers, advances in asymmetrical nucleosome assembly, the emergence of DNA-barcoded libraries, and the use of photo-crosslinking strategies.

At the time of the “2011 perspective” the transition from studying chromatin processes using modified histone-derived peptides to using designer chromatin was still in its early stages. At that time, only a limited number of these substrates had been generated and none of these were commercially available. Thus, in many cases, chromatin biologists did not have access to the specific designer chromatin substrates needed for their work. Correcting this situation was the motivation for writing the “2011 perspective”; it seemed to us imperative that more protein chemists work in this area. From the vantage point of the present day, there seems little doubt that this has happened. Many chemical biologists have been attracted to the field and, collectively, they have contributed mightily to its advancement. This has not only led to the development of robust protocols for the generation of numerous types of designer chromatin each displaying one or more epigenetic marks, but also to the introduction of entirely new chemistry-driven strategies (few of which were anticipated back in 2011) for interrogating chromatin (Fig. 1). Several of these strategies are discussed in the sections below and include the installation of various types of asymmetry into designer chromatin, the incorporation of sophisticated biochemical and biophysical probes into chromatin, and the development of library-based technologies for accelerating biochemical discovery. Importantly, this explosion of work has also made designer chromatin more accessible to the masses since many types of modified histones and MNs are now commercially available.

## Installation, readout, and removal of chromatin modifications

Eukaryotic genomes are packaged into chromatin, the nucleoprotein complex that comprises DNA and histone proteins, wherein the nucleosome serves as the basic repeating unit. Chromatin plays two seemingly opposing functions, stably storing several feet of DNA in the confined space of the nucleus whilst allowing access to specific genomic loci during replication, transcription, and DNA damage repair. These processes are exquisitely regulated meaning that the structure of chromatin, and in particular the positioning and density of nucleosomes, must also be dynamically controlled. It is now well established that chemical modifications (sometimes referred to as “marks”) to both the DNA and histones play a central role in this molecular choreography. Indeed, the collective work of Dave Allis and his many co-workers and collaborators played a pivotal role in elevating histones from mere packaging agents to central players in the regulation of DNA transactions (12, 13, 14, 15). We now know that histones are modified with a truly astonishing number of PTMs (16). Understanding the rules by which these PTMs are installed and removed, as well as what they actually do when present, constitutes a core activity in the molecular epigenetics field. Cell-based studies have proven incredibly powerful in this regard since they provide systems-level information on how these PTMs correlate with, for example, transcriptional activation. However, they typically do not allow direct causal relationships to be drawn. By contrast, such information is possible through controlled *in vitro* biochemical studies employing compositionally defined reconstituted systems. Here, the availability of chemically homogeneous designer chromatin, in which the location of pre-installed PTMs and/or various biochemical probes is known, has been a boon to the community. We illustrate this through select examples below.

### Biochemical crosstalk between histone PTMs

Designer chromatin has played a major role in studying the interplay between different PTMs on chromatin. Proteomics studies have shown that histones can be simultaneously decorated with multiple PTMs, either of the same or different chemotypes (17, 18, 19). Bearing in mind that nucleosomes contain two copies of each of four the core histones H2A, H2B, H3, and H4, then clearly the number of discreet PTM patterns possible on any given nucleosome is extremely large. Adding to the combinatorial complexity is the fact that sister histones in the same nucleosome need not be modified in the same way (20, 21, 22) (see below). While the true number of compositionally unique nucleosomes (at the level of histones) in, for example, human chromatin is a matter of debate, there is no question many nucleosomes contain multiple PTMs on one or more core histones (17, 18, 19). This raises the question of whether the presence of one PTM can influence the installation, readout, or removal of another. In other words, are the writers, readers, and erasers of histone PTMs able to integrate the chromatin landscape as part of their regulation, and if so, how?

**Figure 2:**
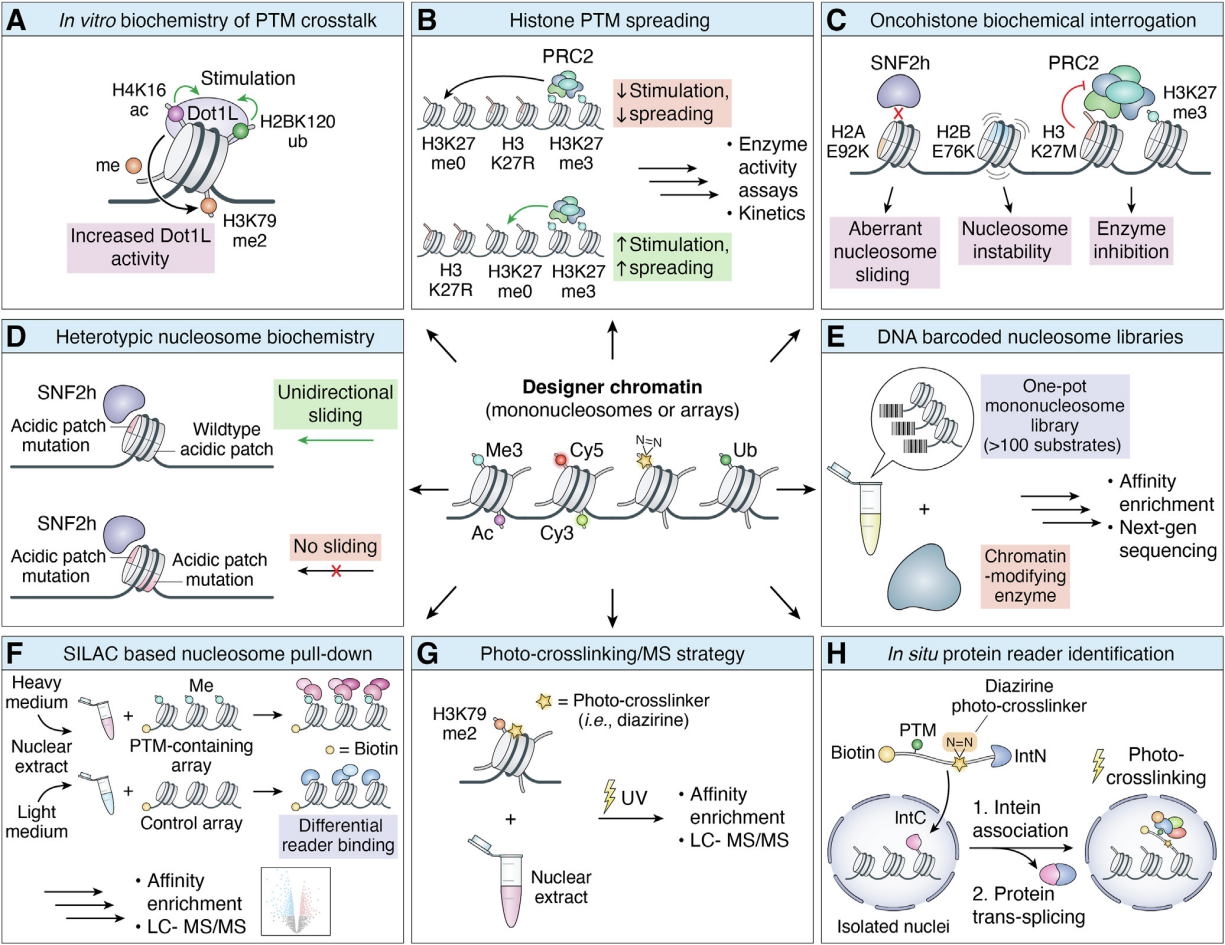
**Applications of Designer Chromatin.***A*, the use of designer nucleosome substrates harboring more than one post-translational modification (PTM) allows for the study of PTM crosstalk. The H3K79me2 methyltransferase, Dot1L has been shown to be stimulated by the presence of both H2BK120ub and H4K16ac. *B*, heterotypic nucleosome blocks containing different configurations of histone PTMs allow for the elucidation of PTM spreading. *C*, reconstituted mononucleosomes containing oncohistone mutations have revealed aberrant enzyme function at key nucleosome regions, such as at H2AE92K which perturbs chromatin remodeling, H2BE76K which increases nucleosome instability and H3K27M which inhibits the methyltransferase, PRC2. *D*, nucleosomes harboring oriented heterotypic copies of acidic patch mutations show differential ISWI remodeling activity depending on which side the acidic patch mutant resides. *E*, DNA barcoded libraries allow one-pot biochemical screening of designer nucleosome substrates. *F*, SILAC (Stable isotope labeling using amino acids in cell culture)-based lysate “nucleosomal dipping” approaches allow for quantitative proteomic analysis of differential chromatin protein binders. *G* and *H*, photo-crosslinking strategies explore chromatin interactomes through quantitative proteomics and identify direct binders to histone PTMs using photo-chemical crosslinking.

Histone-derived peptides provide a convenient vehicle for studying histone PTM crosstalk when the modifications in question are close together in the primary sequence. Indeed, synthetic peptides were key to some of the early, seminal work by the Allis group on the mutually exclusive relationship between trimethylation of lysine nine and phosphorylation of serine ten on histone 3 (15, 23)—work that contributed to the conception of the histone code idea (13). Short synthetic peptides cannot be used to study *trans-*histone PTM crosstalk, the existence of which was suggested by genetic studies indicating dimethylation of Lys79 on H3 (H3K79me2) was dependent on the ubiquitylation of lysine 120 in H2B (H2BK120ub) (24, 25, 26). Whether this dependency operated through a direct or indirect mechanism involving the writer of H3K79me2, Dot1L, was unclear. In work that pre-dates the “2011 perspective,” our group was able to show that designer chromatin containing H2BK120ub directly stimulates the activity of Dot1L to install H3K79me2 (27). In follow-up work, we were able to deduce the mechanistic basis of this crosstalk and demonstrate that H2BK120ub also directly stimulates H3K4 methylation (28, 29, 30, 31, 32, 33). This biochemical work in some ways served as a blueprint for many subsequent studies employing designer chromatin substrates, each documenting *trans*-histone or histone-DNA modification crosstalk (Fig. 2*A*). Those published since the “2011 perspective” include, but are certainly not limited to, the stimulation of the H3K27 methyltransferase, PRC2, by H2AK119ub (34) activation of *de novo* DNA methyltransferases DNMT3a and DNMT3b by H3K36 methylation (35, 36, 37, 38) and the stimulation of the H3K79me2 methyltransferase Dot1L by H4K16 acetylation (39).

Many histone PTM writers and eraser enzymes contain one or more reader domains that can bind to histone PTMs, including the products of their enzymatic activity. This is thought to be essential to the establishment of chromatin states covering multiple nucleosomes through positive or negative feedback mechanisms—a process often referred to as “PTM spreading” (40, 41). Designer chromatin has proven useful in studying such processes by furnishing heterotypic nucleosome arrays in which the modification status of each position is defined (Fig. 2*B*). Substrates of this type have helped shed light on the spreading mechanisms of several enzymes, including Suv39h1 which installs the constitutive heterochromatic mark H3K9me3 (42), and G9a and PRC2 which installs the facultative heterochromatin marks, H3K9me2 and H3K27me3, respectively (43, 44, 45). With respect to PRC2, heterotypic arrays were also instrumental in structural biology campaigns that provided atomic-level details on how the enzyme simultaneously engages multiple nucleosomes (one serving as a substrate and the other an allosteric activator) to propagate the H3K27me3 mark (46). Furthermore, heterotypic arrays have also played a role in studying the function of somatic histone mutations that drive certain pediatric cancers. Arguably the most famous of these, a lysine to methionine mutation at position 27 of histone H3 (H3K27M) (47, 48, 49) was shown in initial studies to act as a pseudo-substrate inhibitor of PRC2 (50, 51). Interestingly, the use of heterotypic arrays revealed that the potency of this inhibition dramatically increases when nucleosomes containing the H2K27M mutant are adjacent to nucleosomes containing H3K27me3 (52). This result implies a link between inhibition and active spreading of the H3K27me3 mark by PRC2. It is worth noting that the involvement of histone mutations in cancer onset and progression was unknown at the time the “2011 perspective” was written. These mutations, dubbed “oncohistones” (another Allis coined term), have emerged as an important sub-field in the chromatin area (reviewed in (53, 54, 55)) As with the analysis of PTMs, designer chromatin has been instrumental in the functional analysis of these mutants (54). Indeed, reconstituted MNs and arrays containing various biochemical and biophysical probes have helped show that oncohistones can; (i) inhibit the activity of histone PTM writers (exemplified by the aforementioned H3K27M), (ii) destabilize the structure of the nucleosome (*e.g*., H2BE76K), and (iii) perturb the activity of ATP-dependent chromatin remodeling enzymes such as SNF2h (*e.g*., H2AE92K) (Fig. 2*C*).

### Janus nucleosomes: asymmetry and chromatin

As alluded to earlier, nucleosomes possess pseudo-twofold symmetry due to the presence of two copies of each of the four core histones. The symmetry is broken when one sister histone in a MN differs from the other, *i.e.* the nucleosome becomes heterotypic at the level of one or more of the histone pairs. Thus, the installation of PTMs, or the presence of histone variants or mutants, all can induce asymmetry, something that has now been widely documented through various proteomics and genomics methods (18, 19, 20, 21, 22, 56, 57, 58, 59). The realization that such asymmetry can exist within chromatin raises the obvious question of whether it impacts the activity of downstream processes. The potential of this is particularly easy to imagine when this asymmetry maps to the structured core of the nucleosome since this would, in effect, lead to the presentation of two different faces on the particle, what we have referred to as Janus nucleosomes (20). Given that many factors engage nucleosomes through facial epitopes, most famously the so-called acidic patch contributed by acidic residues on H2A and H2B (60, 61), it seems possible that chromatin transactions could be altered by such Janus nucleosomes (Fig. 2*D*).

Exploring the role of asymmetry on nucleosomal processes is far from straightforward. Biochemical investigations require access to the necessary substrates which cannot be generated using the standard procedures employed in assembling symmetric designer chromatin. The difficulties here extend beyond the assembly of heterotypic histone octamers since ideally, it would be optimal to deposit these on the template DNA in just one of the two possible orientations. The last several years have seen a flurry of activity in this area. This has led to the development of several approaches for generating asymmetric nucleosomes, ranging from those that employ iterative affinity enrichment from stochastic mixtures to those that use sophisticated engineering strategies to direct the assembly of only the desired products (62, 63, 64, 65, 66, 67). The availability of these substrates has allowed the impact of nucleosome asymmetry on several processes to be evaluated (20). For example, we and others have documented that the activity of ATP-dependent chromatin remodeling enzymes is highly sensitive to imbalances in the chromatin substrate caused by asymmetry (68, 69, 70). Remarkably, the directionality of nucleosome sliding (*i.e.* lateral movement of the histone octamer along the DNA) induced by ISWI and SWI/SNF remodelers (*e.g*. SNF2h) is controllable depending on the orientation of the asymmetric nucleosome on the DNA. Studies of this type suggest that molecular symmetry represents an additional layer of chromatin (dys)regulation, one that warrants additional exploration in the future. More generally, the ability to manufacture asymmetric chromatin illustrates the impressive technical advances that have been made in the designer chromatin area since the writing of the “2011 perspective.”

### Accelerated chromatin biochemistry

Given the wide range of histone PTMs that exist, any systematic *in vitro* analysis of their potential role in mediating chromatin inputs and outputs presents a daunting challenge, at least if one were to take a “one at a time” approach to this. In response to this, several high-throughput strategies have been developed that are designed to speed things up. In the case of histone-derived peptides, both spatially addressable arrays and encoded combinatorial split-pool libraries have been deployed to great effect (71, 72, 73). Of course, the use of peptide-based libraries does suffer from the same constraints noted above, namely the inability to interrogate processes that require a nucleosomal context. Because of this, our lab has developed several versions of DNA-barcoded recombinant nucleosome libraries allowing for the one-pot interrogation of chromatin effector proteins against large collections of modified substrates (70, 74, 75). This *in vitro* technology, which was not anticipated in the “2011 perspective,” harnesses next-generation DNA sequencing (NGS) as a readout of biochemical reactions on a mixture of encoded nucleosomal substrates (Fig. 2*E*). This has several advantages including high sensitivity, the ability to derive quantitative information (*i.e.*, kinetics, binding constants) through analysis of sequencing reads, and the use of multiplexing strategies allowing many thousands of experiments to be conducted at once. Another benefit of this platform is that it involves the use of a mixture of differentially modified nucleosomes, a situation that better mimics the heterogenous nature of native chromatin compared to performing *in vitro* experiments on individual nucleosomes. This versatile platform has been used to interrogate the impact of histone PTMs and oncohistones on the activity of several chromatin factors including histone chaperones, various PTM writers, and ATP-dependent chromatin remodelers (70, 74, 75, 76, 77, 78). As an example of the latter, the substrate preferences of the family of human SWI/SNF (or BAF) remodelers were comprehensively profiled; work that entailed over 25,000 binding and remodeling measurements and that revealed the role of the differential core subunits within each complex in driving the distinct activity signatures observed (77). For example, it was observed that the most abundant subtype of the mSWI/SNF family, canonical BAF (cBAF), has reduced remodeling activity on 70% of the PTM-modified and variant mononucleosome substrates as compared to unmodified mononucleosomes. Strikingly, this reduction in activity was not observed in the other two major forms of mSWI/SNF, PBAF, or ncBAF, which instead showed reduced remodeling activity on 45 and 30%, respectively, of substrates in the library. It is worth noting that the availability of these DNA-barcoded libraries inverts how designer chromatin was initially employed in epigenetics research. Rather than being used to confirm a mechanistic hypothesis previously developed using genetic approaches, these high-throughput screening platforms can instead provide the impetus for follow-up validation studies.

### Gone fishing: designer chromatin combined with MS-based proteomics

The combination of designer chromatin and quantitative proteomics has proven a potent pairing for identifying factors that engage histone PTMs. Here, the designer chromatin is used as bait to pull out direct interactors to the histone PTMs of interest using typically nuclear lysates as input (Fig. 2*F*). Coupling this with an affinity purification step and quantitative mass spectrometry-based proteomics allows for the identification of the bound proteins. This powerful approach was pioneered by the group of Matthias Mann (79, 80, 81), but has been widely adopted by the chromatin community and used to identify factors that engage a large number of chromatin modifications (and combinations thereof) using both MNs and arrays as baits (reviewed in (82, 83)). As an illustration of just how far this methodology has come, Lukauskas *et al*. recently characterized the interactomes of 80 designer dinucleosome chromatin baits (84). This tour-de-force study surveyed designer substrates with unique PTM signatures and linker DNA compositions representing promoter, enhancer, and heterochromatic chromatin regions, and identified over 2000 chromatin binding proteins. Furthermore, by combining large-scale affinity purification and chromatin immunoprecipitation (ChIP)–MS approaches with sophisticated next-generation sequencing (NGS) and proteomic computational methods, the authors were able to quantify chromatin effector protein binding and draw conclusions regarding co-regulated protein interaction networks and complexes unique to each specific histone PTM signature. Through this multi-faceted approach, the authors discovered that many of the chromatin binding proteins detected have broad binding profiles that are not defined by a single chromatin feature. Rather, a combinatorial readout of multi-subunit complexes is often observed. This work highlights how designer chromatin can aid in the development of the governing rules underlying chromatin regulation.

One of the caveats of the above affinity purification approach is that it may fail to identify weakly bound or more transient chromatin interactors. A classic solution to this type of problem has been to employ affinity labeling in which a chemical crosslinker (commonly a photocrosslinker such as a diazirine or benzophenone) is placed close to the binding epitope of interest. Not surprisingly, initial affinity labeling studies in the chromatin area employed histone-derived peptides (85, 86, 87). For example, H3 peptides containing photocrosslinkers were used to show that the H3K27M oncohistone engaged the catalytic subunit of PRC2 (50, 51). Following the familiar trend seen with other applications of chemical biology to chromatin, more recent affinity labeling studies have transitioned to the use of designer chromatin (Fig. 2*G*). In one impressive example of this, Li and coworkers employed a sophisticated photocrosslinking strategy to show that H3K79me2, whose reader protein had long been elusive, specifically binds to the transcription factor, menin (88). Follow-up cryo-EM studies with H3K79me2 nucleosomes revealed that menin interacts with only one face of the nucleosome and recognizes the mark using its finger and palm domains.

Conceivably, the use of nuclear lysates in photo-affinity labeling studies could lead to false positives or negatives given that such inputs are somewhat removed from the normal physiological context. One solution to this is to adapt photo-crosslinking such that it can be performed in a native chromatin context (Fig. 2*H*). This has been achieved using two approaches, genetic code expansion, and split intein-mediated protein trans-splicing. In the former, the unnatural amino acid, in this case, an amino acid bearing the photocrosslinker, is introduced into the target protein in response to (typically) an amber stop codon (6). Both benzophenone and diazirine-based warheads have been introduced into histones using this powerful strategy, allowing photo-crosslinking to be carried out on native chromatin in living cells (89, 90, 91). For example, Kapoor and co-workers employed amber suppression technology to incorporate a diazirine-containing amino acid into histones, allowing covalent capture of chromatin interactors to be characterized as a function of the cell cycle (92). In a complementary approach, our laboratory employed in nucleoprotein trans-splicing technology to install a diazirine photo crosslinker adjacent to various histone PTMs thereby permitting the identification of direct readers of these modifications following quantitative proteomics-MS analysis (93). More recently, protein trans-splicing was combined with the so-called μMap photo-proximity labeling technology (94). The μMap system is based on blue light irradiation of an iridium-centered photocatalyst which leads to the generation of a reactive carbene species derived from an exogenously added aryl-diazirine cell permeable probe (95). Importantly, the labeling radius of the carbene probe is extremely short, ∼10 nm, or on the order of a nucleosome (96). Targeting the photocatalyst to a histone, *via nucleo*protein trans-splicing, revealed how the local interactome of chromatin is altered following various types of cellular perturbation (94). For example, cancer-associated mutations in H2A which map to the acidic patch of the nucleosome were found to displace many epigenetic factors including multiple chromatin remodelers, polycomb subunits, sirtuins, lysine demethylases, and DNA methyltransferases. In follow-up studies, it was demonstrated that this acidic patch onco-mutation leads to an increase in histone H4 acetylation and a decrease in *de novo* DNA methylation, and consequently alters chromatin structure as gauged by ATAC-seq (94).

## Conclusions and future perspectives

In this article, we set out to assess the degree to which chemical biology approaches have been used to tackle problems in chromatin biochemistry, particularly those laid out in the “2011 perspective.” We have focused exclusively on the use of designer chromatin and even here we have covered only a small sampling of what has been done, noting that there is a large body of work using these reagents in structural and biophysical studies that were not reviewed here (see (2, 97)). Nonetheless, we hope that even with the limited examples discussed it will be clear to the reader that the ability to manufacture chromatin using chemistry-driven approaches has been highly impactful. Many chemical biologists now work in this field, something which has led to many adaptations and extensions of the basic designer chromatin platform in ways that far exceed what was envisioned in the “2011 perspective.” As a result of all these innovations, it is now quite commonplace for studies in the epigenetics area to employ designer chromatin at one stage or another. Thus, we think it fair to conclude that the spread of chemical biology into chromatin has been quite pervasive.

This journal issue is dedicated to the late Dave Allis. As pointed out at the beginning of this article, Dave had an uncanny ability to draw scientists into the chromatin field, whether it be through collaborations, simply connecting people, or by communicating the breakthroughs and emerging concepts in the field in ways that were accessible and exciting to a broad audience. A great many people can trace their decision to work in this field based on an encounter with Allis, whether direct or indirect. This legacy has created a stable culture of interdisciplinary research, one that has helped ensure that the type of correlative insights obtained from omics datasets are translated into molecular mechanisms using reconstituted systems. Looking forward, we can imagine that there will be additional synergies and associated innovations involving the use of designer chromatin broadly defined. For example, additional efforts are envisioned that will allow the locus-specific incorporation of histone PTMs and biochemical probes into cellular chromatin, a capability that would open many new opportunities. Additionally, designer chromatin would seem to be well suited to study chromatinized DNA that is extra-chromosomal such as that associated with cancer-associated micronuclei, circular viral genomes, or even Neutrophil extracellular traps. Thus, we expect no letup in the involvement of chemical biologists in all things chromatin, a prospect that doubtless would meet with the enthusiastic approval of Dave Allis.

## Data availability

All data are contained within the manuscript.

## Conflict of interest

The authors declare that they have no conflicts of interest with the contents of this article.
